# Paralytic effect of alcoholic extract of *Allium sativum* and *Piper longum* on liver amphistome, *Gigantocotyle explanatum*

**DOI:** 10.4103/0253-7613.41040

**Published:** 2008

**Authors:** T.U. Singh, D. Kumar, S.K. Tandan

**Affiliations:** Division of Pharmacology and Toxicology, Indian Veterinary Research Institute, Izatnagar - 243 122, UP, India

**Keywords:** Alcoholic extract, *Allium sativum*, *Gigantocotyle explanatum*, *Piper longum*, *spontaneous muscular activity*

## Abstract

**Objective:**

To investigate the effects of alcoholic extract of *Allium sativum* and *Piper longum* on the muscular activity of a parasitic amphistome, *Gigantocotyle explanatum*.

**Materials and Methods:**

Amphistomes were isometrically mounted to record the spontaneous muscular activity by using Chart 4 software program (Power Lab, AD Instruments, Australia) and to examine the effects of cumulative doses (100, 300, 1000, and 3000 μg/ml) of the plant extracts on the amplitude (g), frequency (per 10 min), and baseline tension (g) of the spontaneous muscular activity of the amphistome.

**Results:**

Alcoholic extract of *A. sativum* produced significant reduction in the frequency and amplitude of contractile activity of the amphistome at 1000 and 3000 μg/ml bath concentrations. Complete paralysis of the amphistome was observed after 15 min of addition of 3000 μg/ml concentration. Alcoholic extract of *P. longum* also caused paralysis following 15-20 min exposure of the amphistome to 3000 μg/ml concentration. In both the cases the amphistomes did not recover from paralysis following 2-3 washes.

**Conclusion:**

The observations demonstrate the paralytic effect of alcoholic extract of *A. sativum* and *P. longum* on *G. explanatum*.

Parasitic infection is a major health problem throughout the world and is responsible for considerable economic losses to the livestock industry, particularly to poor livestock owners in developing countries. Other adverse effects of these parasites include loss of meat, wool, and egg production. Amongst helminths, infection caused by trematodes like amphistomes (*Gigantocotyle explanatum* and *Gastrothylax crumenifer*) and *Fasciola* sp. is more serious than that due to round worms. The prevalence of *G. explanatum* is very high in ruminants in the Indian subcontinent. These parasites cause hemorrhages and connective tissue proliferation at the site of attachment, vacuolar degeneration in the liver, and hyperplasia in the bile duct, thereby seriously affecting the health and productivity of infected animals.[1]

Chemotherapy is the only efficient and effective tool to cure and control the helminth infection, as efficacious vaccines against helminths have not been developed so far. Indiscriminate use of synthetic anthelmintics in domestic animals has resulted in the development of resistance in helminth parasites.[2–4] Further, residual toxicity, adverse reactions, high cost, and inaccessibility to the rural farmers are problems associated with these agents. Consequently, there is an urgent need to develop newer, selective, and eco-friendly agents to control helminth infections. Plant-based anthelmintics offer an alternative to overcome some of these problems and they can be both sustainable and environmentally acceptable. Unlike synthetic anthelmintics, plant-based anthelmintics with different modes of action could be of value in preventing the development of resistance.[5] Herbal drugs have been in use since ancient times for the treatment of a variety of acute and chronic parasitic diseases, both in human and in veterinary medicine.[6–7] The use of an extract of male fern (*Dryopteris felix mas*) against cestodes and trematodes and that of arecoline (from *Areca catechu*) against tapeworms of dogs and poultry has also been reported.[8]

In the search for plant-based anthelmintics, extracts of different medicinal plants have been tested for action against flatworms and roundworms *in vitro* and *in vivo* and have been found to possess anthelmintic activity. For example, an alcoholic extract of *Mallotus philippinensis* caused complete paralysis of *F. gigantica in vitro*,[9] garlic protected mice against *Schistosoma* infection, *A. sativum* has shown anthelmintic action *in vitro* against *Heterakis gallinae* and *Ascaridia galli*,[10] *Haemonchus contortus,*[11] a free-living nematode of *Rhabditis* sp., larvae of *Nippostrongylus brasiliensis*, and eggs of *Ascaris summ.*[12] *In vivo* *A. sativum* has demonstrated activity against strongyloids in donkeys.[13] *P. longum* has been reported to produce paralysis of *Ascaris lumbricoides.*[14] Moreover, extracts of the plants used in the present study, *A. sativum* and *P. longum*, have shown good hepatoprotective activity in rats.[15,16] Although *A. sativum* and *P. longum* have shown activity against roundworms, to the best of our knowledge, there is no study on the effects of these plants on *G. explanatum*. The present study evaluates the effects of alcoholic extract of *A. sativum* and *P. longum* on spontaneous muscular activity of *G. explanatum*.

## Materials and Methods

### Collection of parasites

Mature and healthy *G. explanatum* were collected from the bile ducts of freshly slaughtered buffaloes from the local abattoir in warm (38 ± 1°C) Hank's balanced salt solution (HBSS) containing antibiotics (streptomycin sulphate - 6900 units @ 10mg/ml; and benzyl penicillin - 9900 units/liter). They were brought to the laboratory in an insulated container and were kept in the BOD incubator at 38 ± 1°C until further use.

### Preparation of alcoholic extract of the plant material

The plant materials (dried fruits of *P. longum* and bulbs of *A. sativum*) were pounded and then extracted with 70% ethanol under reflux. The alcoholic extract was concentrated under reduced pressure to a semisolid mass and made free from solvent.

### Preparation of plant extract suspension

Suspensions (100 mg/ml) of the alcoholic extracts of *A. sativum* and *P. longum* were prepared in Tween-80 (final concentration 0.1%) and distilled water just before their use and further dilutions were prepared in HBSS solution.

### Isometric mounting of G. explanatum and mechanical recording of the spontaneous muscular activity

Mature and active *G. explanatum* were mounted isometrically in HBSS solution at 38 ± 1°C as per the method described for *Gastrothylax crumenifer.*[17] Briefly, the amphistomes were mounted with the help of two fine steel hooks. One hook was inserted 1-2 mm caudal to the anterior sucker and fixed to the tip of an aeration tube and another hook was inserted through the surface of the acetabulum and connected to the transducer (Power Lab, AD Instruments, Australia).

The isometrically mounted parasite was equilibrated without any tension for 30 min, following which 300 mg tension was applied and spontaneous muscular activity was recorded using Chart 4 software program (Power Lab, AD Instruments, Australia). Control recordings were made for 15 min before the addition of a drug. During the equilibration period, the bath fluid was changed once every 10 min. Three parameters, namely, frequency (total number of contractions in 10 min), amplitude (average of all peaks per 10 min or average tension) of spontaneous muscular contractions, and baseline tension (average of all minimum levels of contractions used for measuring amplitude) of the isometrically mounted *G. explanatum* were measured. Measurements were made for a period of 10 min immediately before the application of a dose of the extract or before a wash following application of a dose.

### Effect of cumulative concentrations of plant extracts on spontaneous muscular activity of isometrically mounted G. explanatum

Cumulative doses (100, 300, 1000, and 3000 *μ*g/ml) of the plant extract were added to the tissue bath with the isometrically mounted amphistome. Each dose was allowed to act for 15 min with concomitant recording of spontaneous muscular activity of the *G. explanatum*. The effects of various concentrations of plant extracts on frequency and amplitude of spontaneous muscular contractions and on the baseline tension of the isometrically mounted *G. explanatum* were recorded and compared with the control recording.

### Statistical analysis

The results are presented as mean ± standard error of mean. To measure the level of significance, one way ANOVA with Tukey's multiple comparison tests were applied.

## Results

### Recording of spontaneous muscular activity (SMA) of G. explanatum

**Figure 1:**
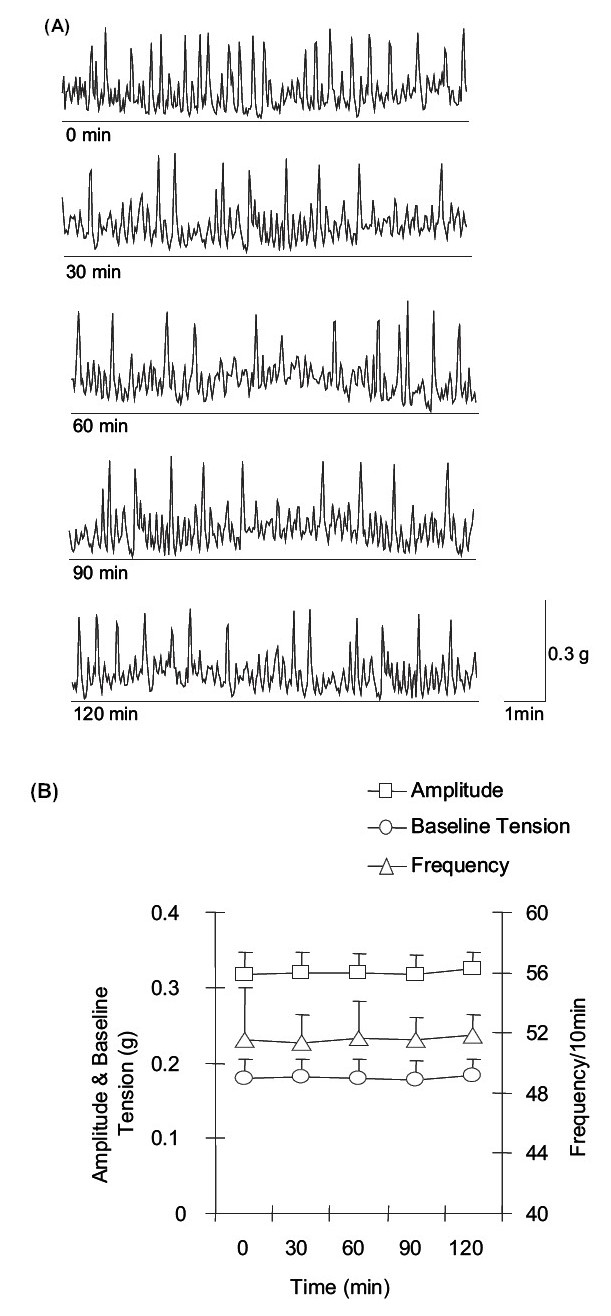
(A) Time-dependent control recording of the spontaneous muscular activity (SMA) of *G. explanatum.* (B) Time-dependent changes in the amplitude (g), baseline tension (g), and frequency (per 10 min) of spontaneous muscular activity (SMA) of *G. explanatum*.

The isometrically mounted *G. explanatum* exhibited SMA for several hours without significant change in amplitude, baseline tension, and frequency of the rhythmicity. The control (i.e., SMA recorded within 15 min of tension application) amplitude, baseline tension, and frequency of SMA were 0.31 ± 0.03 g (*n* = 6), 0.17 ± 0.02 g (*n* = 6), and 51.50 ± 3.52/10 min (*n* = 6), respectively. The amplitude (0.32 ± 0.02 g; *n* = 6), baseline tension (0.18 ± 0.02 g; *n* = 6), and frequency (51.83 ± 1.33/10 min; *n* = 6) of spontaneous contractions recorded after a period of 2 h, were not significantly different from those recorded within 15 min after applying the tension to *G. explanatum* [Figures 1A and B].

### Effect of cumulative concentrations of alcoholic extract of A. sativum on SMA of G. explanatum

**Figure 2:**
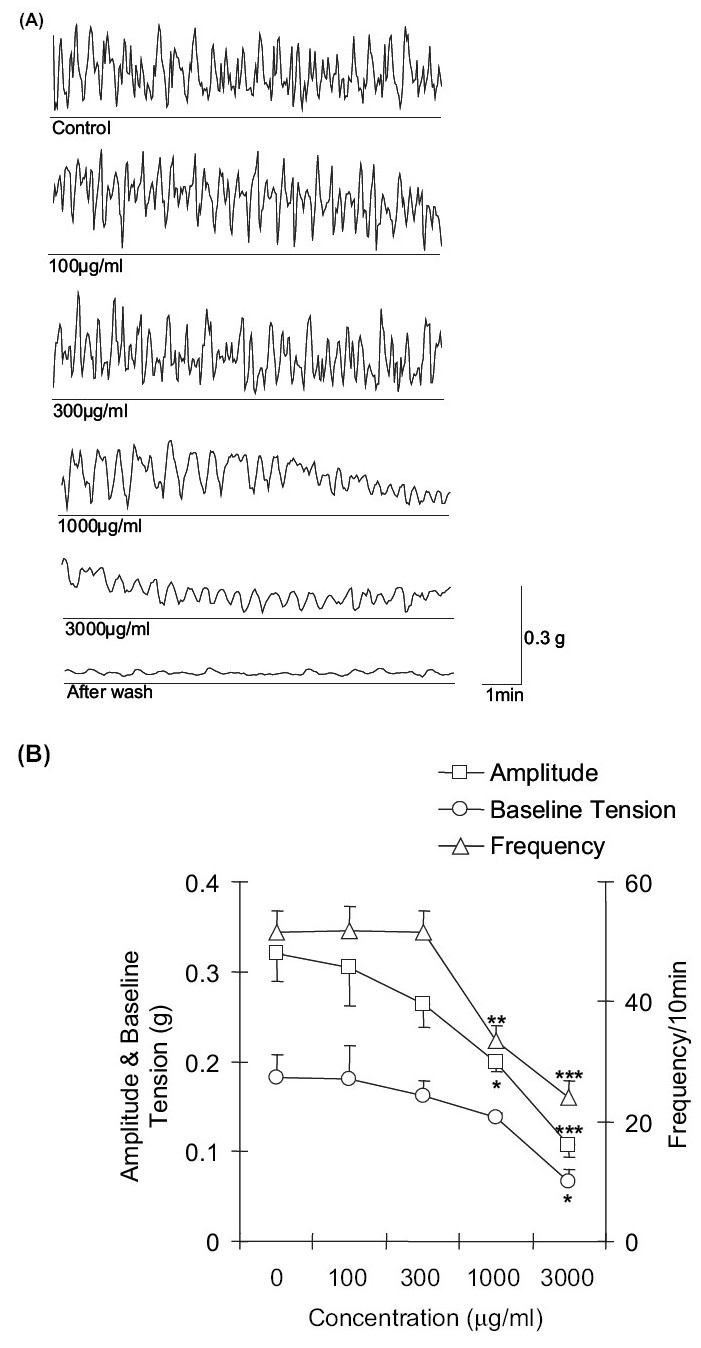
(A) Effect of cumulative concentrations of alcoholic extract of A. *sativum* on spontaneous muscular activity (SMA) of *G. explanatum*. (B) Effect of cumulative concentrations of alcoholic extract of *A. sativum* on the amplitude (g), baseline tension (g), and frequency (per 10 min) of spontaneous muscular activity (SMA) of G. explanatum. Data is expressed as the mean ± SE. *P < 0.05, **P < 0.01 and ***P < 0.001 as compared to control values

The amplitude of contractions of the amphistomes was significantly decreased to 0.20 ± 0.01 g (*P* < 0.05) and 0.10 ± 0.01 g, (*P* < 0.001) with 1000 and 3000 μg/ml concentrations of *A. sativum* extract, respectively, as compared to control (0.32 ± 0.03). The extract, at 1000 and 3000 μg/ml concentrations, also produced significant (*P* < 0.01 and *P* < 0.001, respectively) reduction in the frequency of the SMA of amphistome. The values of frequency of SMA of amphistome at 1000 and 3000 μg/ml concentration of the extract were 33.33 ± 2.73 and 24.00 ± 2.79, respectively. The alcoholic extract of *A. sativum* did not cause any significant change in the baseline tension up to a concentration of 1000 μg/ml. However, at 3000 μg/ml concentration, there was significant (*P* < 0.05) reduction in the baseline tension of the SMA of the amphistome. The extract produced complete paralysis of the fluke after 15-20 min of administration of 3000 μg/ml concentration [Figures 2A and B] and the SMA of the amphistome did not revive following 2 washes at 10 min intervals (data not shown). The recordings demonstrate a dose-dependent and progressive reduction in the SMA of the amphistomes from 100 to 3000 μg/ml concentration of the extract.

### Effect of cumulative concentrations of alcoholic extract of P. longum on the SMA of G. explanatum

**Figure 3:**
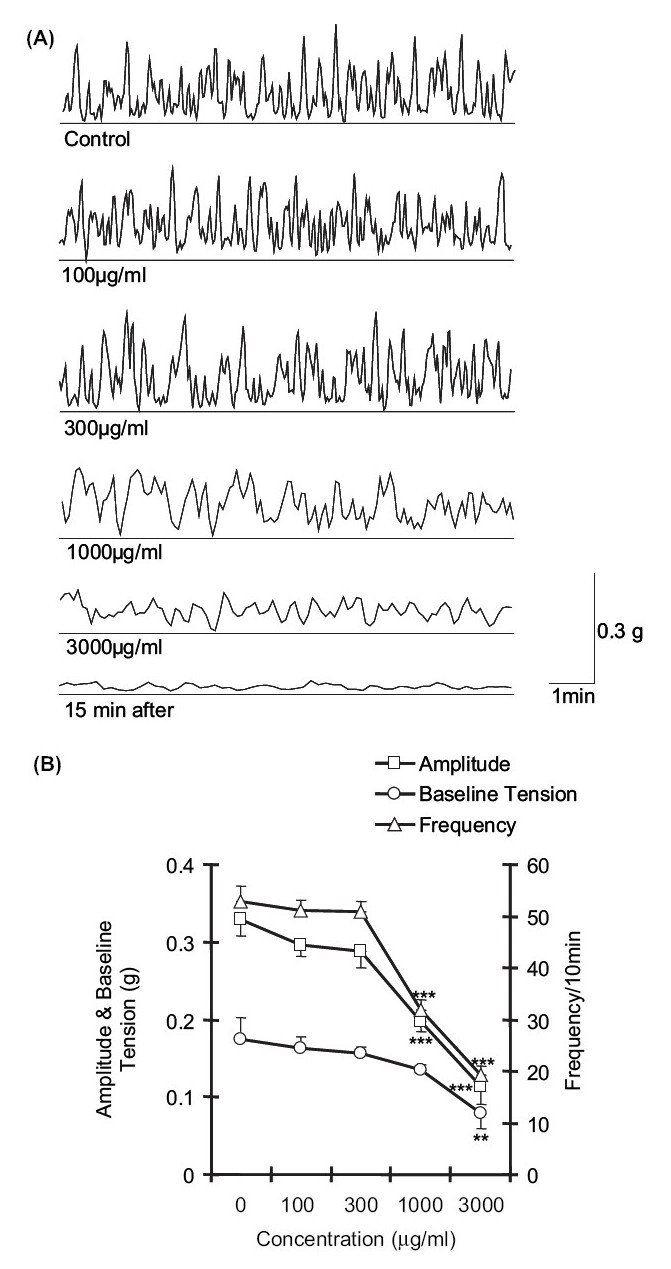
(A) Effect of cumulative concentrations of alcoholic extract of *P. longum* on spontaneous muscular activity (SMA) of *G. explanatum* (B) Effect of cumulative concentrations of alcoholic extract of *P. longum* on the amplitude (g), baseline tension (g), and frequency (per 10 min) of spontaneous muscular activity (SMA) of *G. explanatum*. Data is expressed as the mean ± SE. **P < 0.01 and ***P < 0.001 as compared to control values.

In isometrically mounted *G. explanatum*, the normal amplitude, baseline tension, and frequency of contractions recorded were 0.33 ± 0.02 g (*n* = 6), 0.17 ± 0.02 g (*n* = 6), and 52.83 ± 3.01/10 min (*n* = 6), respectively. The average baseline tension was decreased significantly (*P* < 0.01) with 3000 μg/ml concentration of the extract of *P. longum*. The amplitude was reduced significantly to 0.19 ± 0.01 g (*P* < 0.001) and 0.11 ± 0.02 g (*P* < 0.001) at concentrations of 1000 μg/ml and at 3000 μg/ml, respectively, as compared to control. The frequency of SMA of the amphistome was significantly (*P* < 0.001) reduced at 1000 μg/ml and 3000 μg/ml concentrations of the extract [Figure 3A and B]. Paralysis of the amphistome ensued after 15-20 min of administration of 3000 μg/ml concentration of the alcoholic extract of *P. longum*. The paralyzed amphistome did not revive following 2-3 washes at 10 min intervals.

## Discussion

The main finding from this investigation was that alcoholic extracts of *A. sativum* and *P. longum* produced complete paralysis of *G. explanatum* after 15 min of addition of 3000 μg/ml concentration of the extracts and the amphistomes did not recover from paralysis even after 2-3 washes.

Chemotherapeutic agents available for treatment of helminth infection act mainly through three different mechanisms, viz, by disruption of the neuromuscular physiology, by blocking the energy metabolism, and by disturbing the highly efficient reproductive system of the parasites.[18] Several important anthelmintics cause paralysis of helminth parasites by disrupting one or the other aspect of their neuromuscular system.[8,19]

The muscular activity of helminth parasites can be appreciated as SMA, which can be recorded mechanically with the help of a physiograph. The SMA can be quantified in terms of frequency, amplitude of rhythmic contractions, and baseline tension and these parameters can be measured before and following drug treatment and the values compared.[20] The changes produced in the SMA of isometrically mounted amphistome by a drug, shows the involvement of the neuromuscular system on account of rapidity of action. Rapid and marked change in the SMA of an isometrically mounted parasite by a drug indicates that the neuromuscular system of the parasites has been affected. Thus, SMA of isometrically mounted parasite can be used to evaluate anthelmintic activity of new compounds *in vitro*. In the present study, the SMA of *G. explanatum* is grossly similar to that of *G. crumenifer,*[17] *S. mansoni*,[21] and *F. hepatica.*[22]

We have observed in a separate study that alcoholic extracts of *A. sativum* and *P. longum* inhibited the gross (visually assessed) motility of *G. explanatum* at 4 h of incubation (under publication). In the present study both the extracts induced marked decrease in the amplitude and frequency of rhythmic contractions at 1000 μg/ml concentration and produced complete paralysis of the amphistome within 15 min recording in presence of their highest concentration (3000 μg/ml). It can be appreciated from the recording of the effect of alcoholic extract of *A. sativum* that the inhibitory effect was dose-dependent from 100 to 3000 μg/ml bath concentrations, although same could not be verified statistically. In case of *P. longum* also, a dose-dependent effect could be seen. Alcoholic extract of *P. longum* also demonstrated concentration-dependent inhibitory effect on the SMA of *G. crumenifer* from 1000 to 3000 μg/ml bath concentrations. Thus, the paralysis of *G. explanatum* is the effect of the alcoholic extracts of *A. sativum* and *P. longum*.

The chemotherapeutic value of both the extracts is also evident from an earlier study,[23] wherein *P. longum* (fruits), along with *Azadirachta indica* (bark), *Butea frondosa* (seeds), and *Nigella sativa* (seeds), produced broad spectrum anthelmintic action against roundworms (*H. contortus* and *Oesophagostomum columbianum*) and flukes (*Paramphistomum cervi*) in calves. *In vitro*, *P. longum* produced paralysis of *A. lumbricoides.*[14] Furthermore, *A. sativum* has also been shown to possess anthelmintic action *in vitro* against *H. gallinae* and *A. galli,*[10] *H. contortus,*[11] etc., and *in vivo* against strongyloides in donkeys.[13] The present observations provide evidence for the paralytic effect of alcoholic extracts *A. sativum* and *P. longum* on amphistomes.

In conclusion, the observations demonstrate a paralytic effect of alcoholic extract of *A. sativum* and *P. longum* on *G. explanatum* by progressive reduction in the SMA, which may be associated with their inhibitory effect on the neuromuscular system of the amphistome.

## References

[1] Swarup D, Pachauri SP, Mukherjee SC (1987). Prevalence and clinico-pathology of naturally occurring fascioliasis and biliary amphistomiasis in buffaloes. Indian J Anim Sci.

[2] Singh D, Swarnkar CP, Khan FA (2002). Anthelmintic resistance in gastrointestinal nematodes of livestock in India. J Vet Parasitol.

[3] Von Samson-Himmelstjerna G, Blackhall W (2005). Will technology provide solutions for drug resistance in veterinary helminths?. Vet Parasitol.

[4] Wolstenholme AJ, Fairweather I, Prichard R, von Samson-Himmelstjerna G, Sangster NC (2004). Drug resistance in veterinary helminths. Trends Parasitol.

[5] Hammond JA, Fielding D, Bishop SC (1997). Prospects for plant anthelmintics in tropical veterinary medicine. Vet Res Com.

[6] Chopra RN, Nayyar SL, Chopra IC (1956). Glossary of Indian Medicinal Plants.

[7] Kumar D, Rao GS, Raviprakash V, Tripathi HC, Tandan SK, Lal J (2005). Indigenous plants active against helminthic infections of domestic animals in India. Proc Natl Acad Sci India.

[8] Reinemeyer CR, Courtney CH, Adam HR (2001). Chemotherapy of parasitic diseases. Veterinary Pharmacology and Therapeutics.

[9] Kushwaha DS, Kumar D, Tripathi HC, Tandan SK (2004). Effect of some indigenous medicinal plant extracts on *Fasciola gigantica in vitro*. Indian J Anim Sci.

[10] Nagaich SS (2000). Studies on the anthelmintic activity of *Allium sativum* (Garlic) oil on common poultry worms *Ascaridia galli and Heterakis gallinae*. J Parasitol App Anim Biol.

[11] Zafar-Iqbal, Nadeem QK, Khan MN, Akhtar MS, Waraich FN (2001). *In vitro* anthelmintic activity of *Allium sativum, Zingiber officinale, Cucurbita mexicana* and *Ficus religiosa*. Int J Agric Biol.

[12] Chybowski J (1997). Study of the anthelmintic activity of garlic extracts. Herba Polonica.

[13] Sutton GA, Haik R (1999). Efficacy of garlic as an anthelmintic in donkeys. Israel J Vet Med.

[14] D'-Cruz JL, Nimbkark AY, Kokate CK (1980). Evaluation of fruits of *Piper longum* and leaves of *Adhatoda vasica* nees for anthelmintic activity. Indian Drugs.

[15] El-Manakhy EM, Yaussef SA, Hassieb MM (1998). The protective effect of garlic against carbon tetrachloride-Induced hepatotoxicosis in male rats: Some histopathologic and histochemical studies. Egyptian J Comp Path Clin Pathol.

[16] Rege N, Dhanukar S, Karandikar SM (1984). Hepatoprotective effects of P *longum* against carbon tetrachloride induced liver damage. Indian Drugs.

[17] Noble TG (2004). *In vitro* studies on some medicinal plant extracts against *Gastrothylax crumenifer*, a rumen amphistome.

[18] Geary TG, Klein RD, Vanover L, Bowman JW, Tompson DP (1992). The nervous system of helminths as target for drugs. J Parasitol.

[19] Loukas A, Hotez PJ, Brunton LL, Lazo JS, Parker KL, Goodman, Gilman's (2005). Chemotherapy of helminth infections. The Pharmacological Basis of Therapeutics.

[20] Sukhdeo SC, Sangster NC, Mettrick DF (1986). Effects of cholinergic drugs on longitudinal muscle contraction of Fasciola hepatica. J Parasitol.

[21] Mellin TN, Busch RD, Wang CC, Kath G (1983). Neuropharmacology of the parasitic trematode *Schistosoma mansoni*. Am J Trop Med Hyg.

[22] Fairweather I, Holmes SD, Threadgold LT (1983). *Fasciola hepatica*: A technique for monitoring *in vitro* motility activity. Exp Parasitol.

[23] Raje AA, Jangde CR, Kolte SW (2003). Evaluation of anthelmintic activity of mixture of indigenous medicinal plants in cow calves. J Vet Parasitol.

